# A COVID‐19 model for local authorities of the United Kingdom

**DOI:** 10.1111/rssa.12988

**Published:** 2022-12-13

**Authors:** Swapnil Mishra, James A. Scott, Daniel J. Laydon, Harrison Zhu, Neil M. Ferguson, Samir Bhatt, Seth Flaxman, Axel Gandy

**Affiliations:** ^1^ MRC Centre for Global Infectious Disease Analysis, Abdul Latif Jameel Institute for Disease and Emergency Analytics (J‐IDEA) Imperial College London London UK; ^2^ Department of Mathematics Imperial College London London UK

## Abstract

We propose a new framework to model the COVID‐19 epidemic of the United Kingdom at the local authority level. The model fits within a general framework for semi‐mechanistic Bayesian models of the epidemic based on renewal equations, with some important innovations, including a random walk modelling the reproduction number, incorporating information from different sources, including surveys to estimate the time‐varying proportion of infections that lead to reported cases or deaths, and modelling the underlying infections as latent random variables. The model is designed to be updated daily using publicly available data. We envisage the model to be useful for now‐casting and short‐term projections of the epidemic as well as estimating historical trends. The model fits are available on a public website: https://imperialcollegelondon.github.io/covid19local. The model is currently being used by the Scottish government to inform their interventions.

## 1 INTRODUCTION

Surveillance systems are vital to combat the spread of the SARS‐CoV‐2 epidemic. In the United Kingdom, publicly available estimates of current infections and the reproduction number (i.e. the number of secondary infections per infection) are primarily available at national and regional level (nine geographic regions of England) (Department of Health and Social Care, 2020). However, with an evolving epidemic localised trends at sub‐national level are very important. To identify trends and facilitate monitoring at a local level, we implement a semi‐mechanistic Bayesian transmission model for SARS‐CoV‐2 at the local authority (LA) level for the United Kingdom. The model assesses and projects the evolution of the epidemic and estimates the time‐varying reproduction number for local areas. We apply our analysis to the United Kingdom, although our approach is applicable to any country where local data on cases and deaths are available.

We have extended the Flaxman et al. (2020) model for each local authority by incorporating four innovations. First, the model incorporates reported cases in addition to deaths. Second, survey data from the (Office for National Statistics, 2020a) and from the Real‐time Assessment of Community Transmission study (REACT Study, 2020) is used to calibrate estimates of the (unobserved) number of true infections. Third, the model incorporates a time‐varying infection fatality rate (IFR), the fraction of infections that leads to deaths, and a time‐varying infection ascertainment rate (IAR), the fraction of infections identified as positive cases. Fourth, infections are modelled as a random process, and not merely a deterministic function of $R_{t}$, previous infections, and a given serial‐interval (generation distribution), which better accounts for variability in areas with low infection numbers.

Regularly updated results from the model are presented at covid19local.^1^The model is currently being used by the Scottish government in their response to their epidemic (Scottish Government, 2020, issue 24 to now).

## 2 DATA

We combine data from national statistics and public health bodies across the United Kingdom. Reported cases for England are taken from the UK Coronavirus dashboard (Public Health England, 2020), for Wales from (Public Health Wales, 2020). For England and Wales, information about deaths are from (Office for National Statistics, 2020b). Cases and deaths for Northern Ireland are from (Department of Health, Northern Ireland, 2020) and for Scotland are from (Scottish Health and Social Care Open Data, 2020).

For reported cases, the model uses the date of specimen collection. To account for reporting variations within a given week, we aggregate daily case and death data by week. We omit the last 3 days of data while fitting our model to account for reporting delays. ONS and REACT survey data are used to calibrate estimates of infections produced by our model.

Our model works at the local authority level—in England, where there are upper and lower tier local authorities (LTLAs), we work with LTLAs. The other nations of the United Kingdom do not have this subdivision. In total our model works with 391 different areas, that consists of the United Kingdom, nations in the United Kingdom, geographic regions of England and all local areas in the United Kingdom.

## 3 MODEL

Flaxman et al. (2020) introduced a Bayesian semi‐mechanistic framework for estimating the transmission intensity of SARS‐CoV‐2. The model is based on the renewal equation (Mishra et al., 2020), and uses $R_{t}$ to generate new infections. We modify this framework for use with local authorities. In this section, we outline the model; more details are in the supplementary materials. Section 6 discusses the reasons for our modelling choices.

Let $i_{t}$ be the number of infections on a given day t in a given area. The basic model in (Flaxman et al., 2020) uses the following renewal equation:

(1) $$i_{t}=R_{t}\overset{t-1}{\underset{\tau =0}{\sum }}\;i_{\tau }g_{t-\tau },$$

where $R_{t}$ is the real‐time reproduction number (i.e. the number of secondary infections per infection), and $g_{k}$, k=1,… is the generation distribution, that is, a probability mass function determining the time between two infections. $R_{t}$ is random and can be flexibly modelled.

We use a modified version of (1) in our models, to allow for both randomness in the infection process and for population effects such as a decreasing susceptible population over time (Bhatt et al., 2020; Scott et al., 2021). We denote by $i_{t}$ the number of infections at time t. Based on the infections up to time t−1 given by $i_{0},\ldots ,i_{t-1}$ (which include population effects), we first model $i_{t}^{\prime }$, the number of infections that would have occurred at time t if everyone was susceptible, as

$$i_{t}^{\prime }∼LN(R_{t}\overset{t-1}{\underset{\tau =0}{\sum }}\;i_{\tau }g_{t-\tau },d),$$

where LN(μ,d) is a log‐normal distribution with the unconventional parameterisation of a mean μ and a SD of $d\sqrt{\mu }$, with d being assigned a prior. This is intended to be a continuous approximation of an overdispersed Poisson distribution, where the parameter d controls the amount of overdispersion.

To account for previous infections and population effects, $i_{t}^{\prime }$ is then modified. We assume a differential equation for the infections during [t−1,t], to avoid discrete time effects such as infections going above the total population. Specifically, we assume that the number of infections i(s) in [t−1,t−1+s) are given by $\partial i(s)/\partial s=i_{t}^{\prime }(1-(\sum_{j=0}^{t-1}i_{j}+i(s))/N)$, where N is the population size. This adjusts $i_{t}^{\prime }$ to account for the continuously decreasing susceptible population. The differential equation has the solution $i(1)=i_{t}$ given by

(2) $$\begin{array}{ll} i_{t}=(N-\overset{t-1}{\underset{j=0}{\sum }}\;i_{j})\left(1-\exp\left(-\frac{i_{t}^{\prime }}{N}\right)\right), & \end{array}$$

which defines the number of infections at time t in our model. When we report reproduction numbers we adjust these for the population effect and report $R_{t}(1-(\sum_{j=0}^{t-1}i_{j})/N)$.

The model contains different observation types (cases, deaths, survey data), each of which will only be present at some time points (e.g. once per week). For an observation of type l at time t, we model the expected observation $o_{t}^{l}$ as a weighted sum of past infections:

(3) $$\begin{array}{ll} o_{t}^{l}=\alpha (t)\overset{t-1}{\underset{\tau =0}{\sum }}\;i_{\tau }\pi_{t-\tau }. & \end{array}$$

The observed data $O_{t}^{l}$ are then noisy versions of this, that is, $O_{t}^{l}∼{ℱ}^{l}\left(o_{t}^{l},\phi \right)$, where ℱ is a distribution (e.g. Poisson, negative binomial) parameterised by its mean $o_{t}^{l}$ and possibly an auxiliary parameter ϕ.

Typical examples for observations include case or death counts. α(t) represents an ascertainment rate and $\pi_{t-\tau }$ represents the distribution that provides the weighting for past infections. For case or death data, α(t) would be the IAR or the IFR, respectively, and $\pi_{t-\tau }$ would be the infection to case or the infection to death distribution, respectively.

In our specific case, we parameterise ℱ as a negative‐binomial distribution for modelling weekly deaths and cases data. To model seroprevalence data we parameterise ℱ as a normal distribution. The exact details on how each different observation is modelled is given in supplementary information.

Interventions are not explicitly included in local models. Instead, we parameterise $R_{t}$ with a random effect for each week of the epidemic, and for each LA separately (no joint inference of parameters across different local authorities). Weekly random effects are encoded as a random walk with normally distributed updates. Thus, under the prior, each successive step the random effect has an equal chance of moving upwards or downwards from its current value. Our model is implemented using epidemia^2^ (Scott et al., 2021), a general purpose R package for semi‐mechanistic Bayesian modelling of epidemics built on top of RStan (Carpenter et al., 2017) and using an interface based on rstanarm (Goodrich et al., 2020).

We take a three‐stage, top‐down approach to fitting LA models.

**FIGURE 1:**
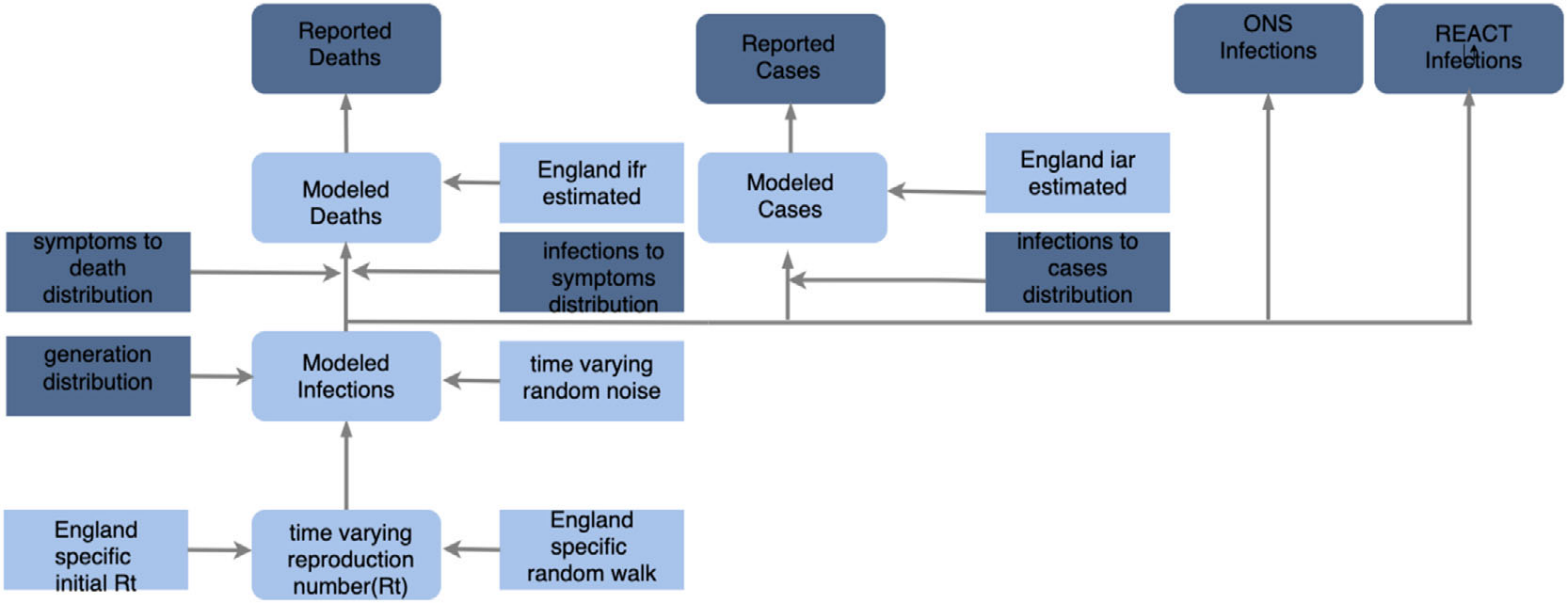
Model diagram for first stage when we have all four different observations (deaths, cases, ONS infections, REACT infections) available for England and we use this to estimate time‐varying infection fatality rate and infection ascertainment rate. Dark blue nodes are observed. [Colour figure can be viewed at wileyonlinelibrary.com]

**FIGURE 2:**
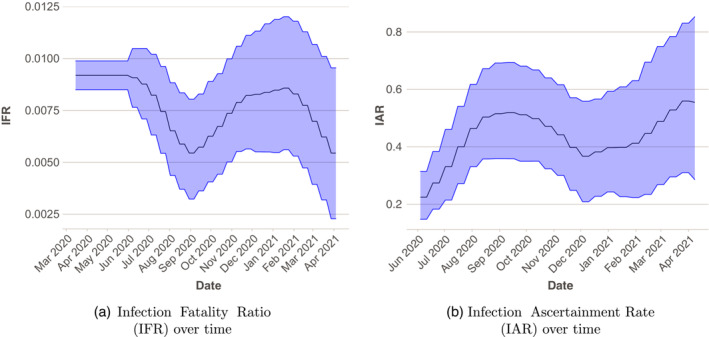
Time‐varying estimates of the infection fatality ratio (IFR) (a), and the infection ascertainment rate (IAR) (b), for England. The solid line is the mean estimate and the filled area denotes 90% pointwise credible intervals. [Colour figure can be viewed at wileyonlinelibrary.com]

We first calibrate the IFR (IFR) and IAR (IAR) by fitting the model at national level (i.e. not broken down by region) to weekly deaths and cases in England, as well as to survey data from ONS and REACT. Figure 1 gives an overview of this stage. For all subsequent individual models, we use these estimated IFR and IAR values (Figure 2) as priors.

In the second stage, we fit individual models to Northern Ireland, Scotland and Wales and to the nine regions of England using death and case data aggregated to regional level from local authority level, using the priors estimated for the IFR and IAR in the first stage. The estimates from these 12 regions provide underlying trends of $R_{t}$ for local areas within a particular region. We report the results of these regions, together with estimates for England and the United Kingdom as a whole. A diagram for this stage is in Figure S1.

Third, we fit individual models for each local area. Local values of $R_{t}$ are parameterised as a sum of a weekly random walk and the estimated value of $R_{t}$ from the region each local area is located in (multiplied by a tight prior around 1). There are three broad components to the model likelihood, arising from cases, deaths and seroprevalence. All model code is available at https://github.com/ImperialCollegeLondon/covid19uklocal, and detailed description of the model and fitting procedure are given in the supplementary material and in Bhatt et al. (2020); Scott et al. (2021). A diagram for this stage is in Figure S2.

## 4 RESULTS

In this section we give some example outputs from the model.

**FIGURE 3:**
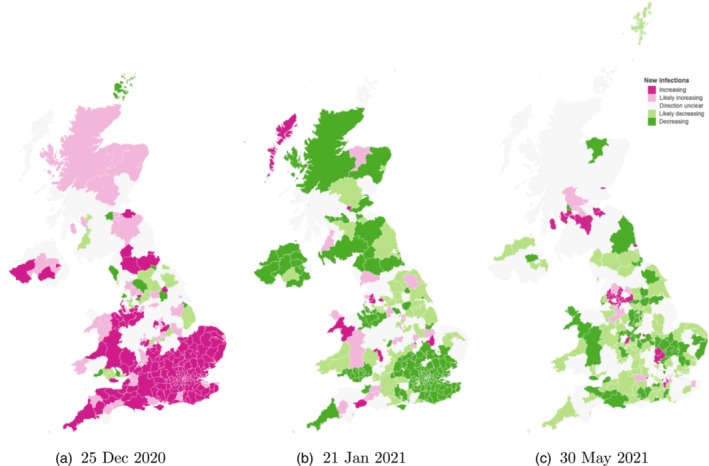
Probability of epidemic growth by local area. We consider infections within a local area to be increasing if $R_{t}>1$ with probability ≥ 90% (dark pink), and likely increasing if $R_{t}>1$ with probability between 75% and 90% (light pink). Decreasing (dark green) and likely decreasing (light green) are defined symmetrically with $R_{t}<1$. [Colour figure can be viewed at wileyonlinelibrary.com]

Figure 3 shows the posterior probability that $R_{t}$ is greater than 1 by local area for three different periods of epidemics, 25 December 2020, immediately before England was put into a national lockdown, 21 January 2021 just after the third lockdown, and 30 May 2021 3 weeks before the planned removal of all restrictions. We include estimates of $R_{t}$ and infections over time for each local area on our website (Gandy & Mishra, 2021), which is updated daily.

**FIGURE 4:**
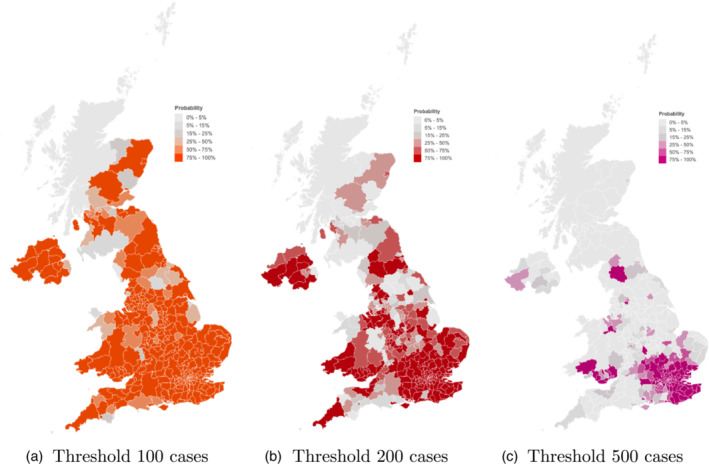
P(hotspot x) for different thresholds. Probability of local authority areas exceeding 100, 200 or 500 cases per 100K population for the period 27 December 2020 to 2 January 2021 as projected on 25 December 2020. [Colour figure can be viewed at wileyonlinelibrary.com]

On our website, we have used the term “hotspot x” (e.g. “hotspot 50,” “hotspot 100”) which we define as a local authority, whose weekly reported cases per 100k population exceed x. We specifically project the probability of an area being a “hotspot x” in the next 3 weeks and report maps of these probabilities. Examples of such maps are shown in Figure 4 for the period 27 December 2020 to 2 January 2021 as projected on 25 December 2020. As on our website, we have chosen different colours for different thresholds to set the plot for different thresholds clearly apart.

A monitoring system could use these probability projections to identify areas of concerns. They offer a combined view of both the current infections as well as the current growth of the epidemic ($R_{t}$) in a single number. A simplistic view that looks at either value in isolation can be misleading: for example, a low case count with high $R_{t}$, through exponential growth, can lead to a sudden increase in infections which can be more concerning than a high case count with (very) low $R_{t}$.

The tier monitoring system in Scotland used output from our model from 22 October 2020 onwards, specifically the hotspot probabilities (Scottish Government, 2020). Initially, our model was used on its own, from 25 February 2021 the outputs of this model were combined with the output from two other models.

## 5 MODEL EVALUATION

**FIGURE 5:**
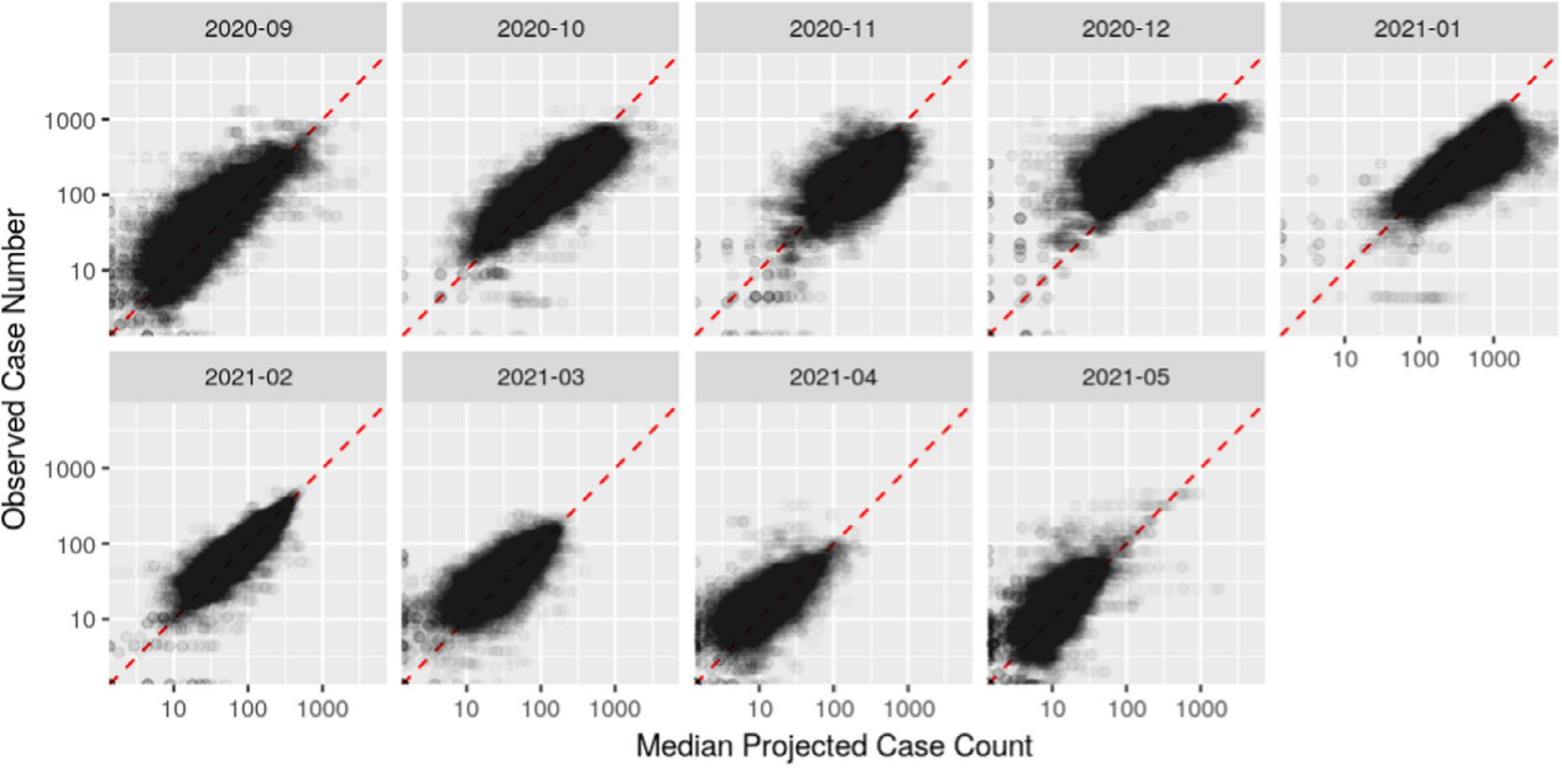
Median projections versus observed weekly case numbers per 100k by month in which the projection was made. [Colour figure can be viewed at wileyonlinelibrary.com]

The model has been run regularly, mostly daily, since September 2020 and results were made publicly available. Projections were made for the upcoming three weeks. Figure 5 shows a comparison of the projections made by the model and the reported weekly case numbers per 100k population. The comparison is by month in which the projection was made. This is an out‐of‐sample test, as all of these projections were made before the case numbers were available.

Figure 5 shows a reasonable correspondence between projections and reported case numbers, in the sense that they seem to be correctly centred. Exceptions are particularly in December 2020 and in January 2021. In December 2020, projections were generally lower than reality—this is most likely due to the emergence of the alpha variant of the virus, which has a substantially increased transmissibility (Volz et al., 2021). In January 2021 the projections were higher than the reported case counts—due to interventions being put into place nationwide. This is expected behaviour from the model—it projects the current state of the epidemic forward—it does not attempt to estimate the effect of policy changes or the emergence of a new variant of the virus.

## 6 DISCUSSION

Our modelling approach has some underlying limitations. We have assumed homogeneous mixing of the population within local authorities and various age groups. All of our probability distributions for the delay between infection and symptom onset, between symptom onset and death, time between a person's infection and their subsequent transmission are assumed to be constant throughout.

Projections from the model assume no change in governmental interventions and human behaviour. Furthermore, interventions are not explicitly included in our models. Hence, the effect of a measure may not appear for 1–2 weeks after its implementation, once the random walk in $R_{t}$ starts picking up a signal in the data. There are several reasons for this approach. First, reliable data on interventions and mobility is typically not immediately available. Even if it were available, the effects on transmission may only be identifiable over time. The random walk in $R_{t}$ can pick up these effects automatically. Second, including interventions in the model would require frequent adjustments to the model, and would make providing daily updates difficult. A side effect of not including interventions is that potentially sharp transitions in $R_{t}$ due to measures may be smoothed over time by the random walk.

An alternative formulation to the model would have the reproduction number R in (1) and/or the ascertainment rates α in (3) depending on the time of infection τ instead of the current time t. We chose the dependence on the current time t, as this allows the model to adapt to changes such as increased testing or non‐pharmaceutical interventions that affect all infected individuals.

Apart from the estimates used from previous steps (e.g. IFR, IAR), we deliberately did not construct a joint model of all regions (e.g. using partial pooling in the spirit of Gelman (2006)). One reason is the computational demand for running such a model. A second reason is that we wanted the projections for one area to be not strongly affected by neighbouring areas, ensuring that decisions for an areas can be justified mostly by information from the same area.

The three stage modelling approach helps to robustly estimate the epidemic. The estimates of IFR and IAR from the first stage are reliable for England as they are inferred using serosurveys from both the ONS and REACT. In all remaining models, the individual IFR and IAR are specified by tight priors with the mean equal to the IFR and IAR for the whole of England. This step permits variation between individual models while also calibrating IFR/IAR against serosurveys. Recall that in the second stage we fit models for all regions in England, as well as for all nations in the United Kingdom. The LA models are then fit using the regional $R_{t}$ (region for LTLAs in England and nations for local authorities in other nations) as a covariate for the local $R_{t}$ in addition to the weekly random walk. The regional $R_{t}$ trend helps to stabilise inference for local areas. Background regional $R_{t}$ values are not used for the last 45 days to ensure that recent trends in local $R_{t}$ are driven primarily by the data from the local area.

The renewal equation (1) propagates infections deterministically. This is generally suitable as infections become large, but in low incidence settings, estimation of $R_{t}$ can be sensitive to random fluctuations and noise. This is why we treat infections as latent parameters which must be sampled. Infections are assigned a distribution with mean given by (1) and coefficient of variation d, which is assigned a prior. This extension reflects a belief that changes in the number of infections at low infection counts provide limited evidence to ascertain $R_{t}$, and must be treated with caution. Equation (2) gives exact details of the formulation.

An important aspect of our framework is the projection of hotspots for all local areas. We argue that for understanding the true state of an epidemic in an area, only specifying $R_{t}$ or current incidence is not enough. Summarising areas with $R_{t}$ can be misleading as areas with low incidence might still be controlled with moderate measures. Similarly, high incidence areas that have $R_{t}<1$ show signs of control and are not in an exponential growth phase. For these reasons, we believe that projections of future cases provide a more relevant indicator. Case projections combine the rate of change and the absolute values of infections into a single actionable number. Moreover, it is key to measure not just projected case counts but also the confidence the model has that those projections will be above a given threshold. Hence, in our framework we define hotspots based on the probability of a projection being greater than a specific threshold.

## Supporting information




**Data S1**. Supporting information

## Footnotes

1
https://imperialcollegelondon.github.io/covid19local

2
https://imperialcollegelondon.github.io/epidemia
