# Supplementary material for: A COVID‐19 model for local authorities of the United Kingdom
Source: J R Stat Soc Ser A Stat Soc. 2022 Dec 13;185(Suppl 1):S86–95. doi: 10.1111/rssa.12988 (PMC9877769; doi:10.1111/rssa.12988)
Supplement: Supplementary file 1 — Data S1. Supporting information [file RSSA-185-S86-s001.pdf]

A COVID-19 Model for Local Authorities of the United Kingdom - Supplementary Material

Swapnil Mishra[1], James A. Scott[2], Daniel J. Laydon[1], Harrison Zhu[2], Neil M. Ferguson[2], Samir Bhatt[1], Seth Flaxman[2], Axel Gandy[2]

[1] *MRC Centre for Global Infectious Disease Analysis, Abdul Latif Jameel Institute for Disease and Emergency Analytics (J-IDEA), Imperial College London, London, UK*

[2] *Department of Mathematics, Imperial College London, London, UK*

E-Mail: a.gandy@imperial.ac.uk

We provide a compact description of the the model. More details are available in Bhatt et al. (2020).

Both deaths and cases are observed in our model. We define weekly deaths, D_w, for weeks $w \in \{1, \ldots, w(n)\}$, where n is the total number of days in the data. These weekly deaths are modelled using a positive real-valued function $d_w = \mathbb{E}[D_w]$ that represents the expected number of deaths attributed to COVID-19. The weekly deaths D_w are assumed to follow a negative binomial distribution with mean d_w and variance $d_w + \frac{d_w^2}{\psi_1}$, where ψ_1 follows a positive half normal distribution, i.e.

$$D_w \sim \text{Negative Binomial}\left(d_w, d_w + \frac{d_w^2}{\psi_1}\right), \quad t = 1, \ldots, n \tag{1}$$

$$\psi_1 \sim \mathcal{N}^+(0, 40). \tag{2}$$

Here, $\mathcal{N}(\mu, \sigma)$ denotes a normal distribution with mean μ and standard deviation σ. We say that X follows a positive half normal distribution $\mathcal{N}^+(0, \sigma)$ if $X \sim |Y|$, where $Y \sim \mathcal{N}(0, \sigma)$.

We link our observed deaths mechanistically to transmission as in Flaxman et al. (2020). We use a previously estimated COVID-19 specific distribution of times from infection to death π, as detailed in Verity et al. (2020); Walker et al. (2020). We assume the distribution of times from infection to death π (infection-to-death) to be the convolution of an infection-to-onset distribution (π') (Walker et al., 2020) and an onset-to-death distribution (Verity et al., 2020):

$$\pi \sim \text{Gamma}(5.1, 0.86) + \text{Gamma}(17.8, 0.45). \tag{3}$$

Similar to generation distribution, π is further discretized via $\pi_s = \int_{s-0.5}^{s+0.5} \pi(\tau) d\tau$ for $s = 2, 3, ...,$ and $\pi_1 = \int_0^{1.5} \pi(\tau) d\tau$, where $\pi(\tau)$ is the density of π.

For estimating time varying infection fatality ratio (IFR, probability of death given infection) we use the estimates from Riley et al. (2020) as a starting point and then a bi-weekly change is estimated using the infections survey data from Office for National Statistics (2020) and REACT Study (2020).

The expected number of deaths d_w, in a given week w, is given by the following discrete sum:

$$d_w = \sum_{\tau=0}^{t-1} \mathrm{IFR}(w) i_\tau \pi^W_{t-\tau}, \tag{4}$$

where t is the total number of days till week w, i_τ is the number of new infections on day τ and where π^W is the transformation of discretized π to a weekly lag.

We also observe weekly cases C_w from 1st June 2020 $w \in \{w(t = \text{2020-06-01}), \ldots, w(n)\}$. Similar to weekly deaths, weekly cases are modelled using a positive real-valued function $c_w = \mathbb{E}[C_w]$ that represents the expected number of cases identified in testing for COVID-19. Again, the weekly cases C_w are assumed to follow a negative binomial distribution but with mean c_w and variance $c_w + \frac{c_w^2}{\psi_2}$, where ψ_2 follows a positive half normal distribution, i.e.

$$C_w \sim \text{Negative Binomial}\left(c_w, c_w + \frac{c_t^2}{\psi_2}\right), \quad t = t_c, \ldots, n, \tag{5}$$

$$\psi_2 \sim \mathcal{N}^+(0, 40). \tag{6}$$

We assume the lag between an infection to be identified as a case, π^{i2c}, is zero for first three days and then has an equal chance over next 10 days, i.e.

$$\pi^{i2c} = c\left(0, 0, 0, repeat(\frac{1}{10}, 10)\right). \tag{7}$$

We link the observed weekly cases and estimated daily infections using an estimated parameter infection ascertainment ratio (IAR), which is defined as the number of reported cases divided by the true number of infections (including both symptomatic and asymptomatic infections).

The expected number of cases c_w, in a given week w is given by the following discrete sum:

$$c_w = \sum_{\tau=t-13}^{t-1} \mathrm{IAR}(w) i_\tau \pi^{i2c}_{t-\tau}, \tag{8}$$

where, again, where t is the total number of days till week w, and i_τ is the number of new infections on day τ.

We also observe daily infections from ONS I_t^{ons} in our model. ONS infections are modelled using a positive real-valued function $i_t^{ons} = \mathbb{E}[I_t^{ons}]$ that represents the expected number of infections given by the ONS infection survey. Daily infections from ONS I_t^{ons} are assumed to follow a normal distribution but with mean i_t^{ons} and standard deviation given by the 95% CI in ONS infection survey, i.e.

$$I_t^{ons} \sim \mathcal{N}\left(i_t^{ons}, \frac{95\%CI}{5}\right), \quad t = t_c, \ldots, n, \tag{9}$$

$$\tag{10}$$

The expected number of ONS infections i_t^{ons}, on a given day t is given :

$$i_t^{ons} = i_t, \tag{11}$$

We also observe total infections from REACT $I_{t,total}^{react}$ in our model. REACT infections are modelled using a positive real-valued function $i_{t,total}^{react} = \mathbb{E}[I_{t,total}^{react}]$ that represents the expected total number of infections given by the REACT. Total infections from REACT $I_{t,total}^{react}$ are assumed to follow a normal distribution but with mean $i_{t,total}^{react}$ and standard deviation given by the 95% CI in REACT survey, i.e.

$$I_{t,total}^{react} \sim \mathcal{N}\left(i_{t,total}^{react}, \frac{95\%CI}{5}\right), \quad t = t_c, \dots, n, \tag{12}$$

$$\tag{13}$$

The expected number of total infections $i_{t,total}^{react}$, on a given day t is given :

$$i_{t,total}^{react} = \sum_{\tau=0}^{t} i_\tau, \tag{14}$$

We parametrise $R_{t,m}$ with a random effect for each week of the epidemic as follows

$$R_t = R_0 \cdot f\left(-\epsilon_{w(t)}\right), \tag{15}$$

where $f(x) = 2\exp(x)/(1+\exp(x))$ is twice the inverse logit function and $\epsilon_{w(t)}$ is a weekly random walk (RW) process, that captures variation between R_t in each subsequent week.

Following Liu et al. (2020), the prior distribution for R_0 was chosen to be

$$R_0 \sim \mathcal{N}(3.28, 0.5) \tag{16}$$

We assume that seeding of new infections begins 30 days before the day after a state has cumulatively observed 10 deaths. From this date, we seed our model with 6 sequential days of an equal number of infections: $i_1 = \cdots = i_6 \sim \text{Exponential}(\frac{1}{\tau})$, where $\tau \sim \text{Exponential}(0.03)$. These seed infections are inferred in our Bayesian posterior distribution.

The weekly effect is modelled as a weekly random walk process, centred around 0 with variance σ_ϵ that, starts on the first day of its seeding of infections, i.e. 30 days before a total of 10 cumulative deaths have been observed in this state. The RW process starts with $\epsilon_1 = 0$,

$$\vec{\epsilon}_{raw} \sim \mathcal{N}(0, 1) \tag{17}$$

$$\epsilon_{w(t)} = \left(\sum_{i=1}^{|\vec{\epsilon}_{raw}|} \epsilon_{raw}\,[i]\right) * \sigma_\epsilon \text{ for } w(t) = 2, 3, 4, \dots \tag{18}$$

The prior for σ_ϵ, the variance of RW process ϵ, is chosen as $\sigma_\epsilon \sim \mathcal{N}^+(0, .2)$. The conversion from days to weeks is encoded in $w(t)$. Every 7 days, w is incremented, i.e. we set $w(t) = \lfloor (t - t^{\text{start}})/7 \rfloor + 1$, where t^{start} is the first day of seeding.

We estimated parameters independently for each local authority. Fitting was performed with the R package epidemia (Scott et al., 2021), written in the probabilistic programming language Stan (Carpenter et al., 2017) using an adaptive Hamiltonian Monte Carlo (HMC) sampler.

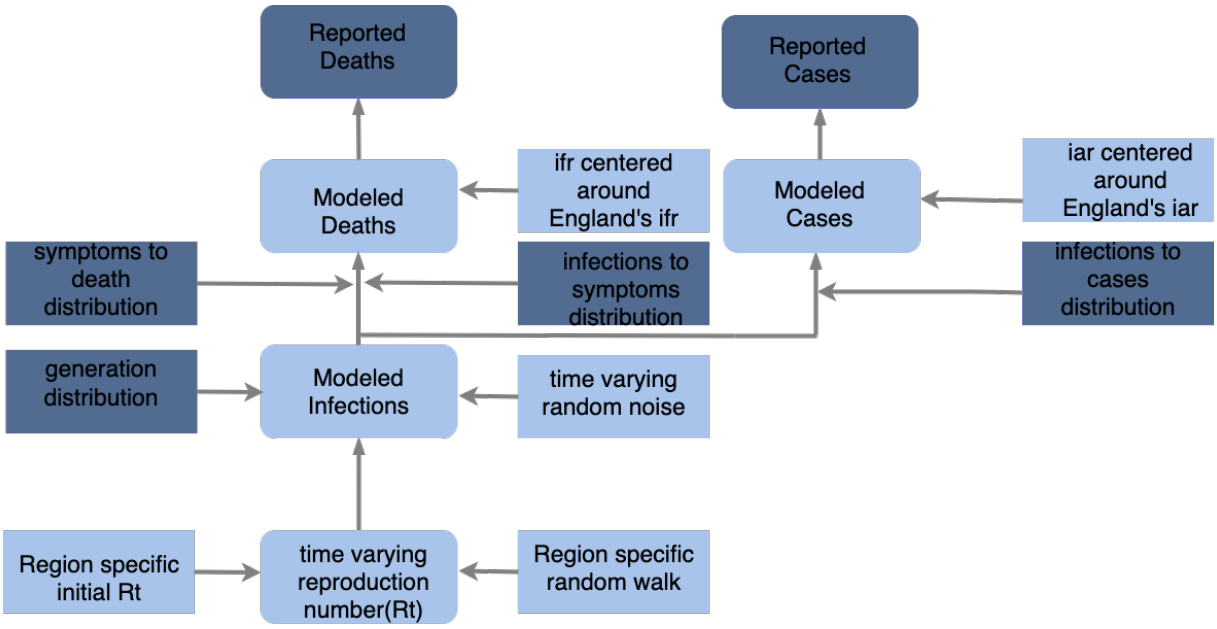

Fig. 1. Model diagram for second stage for regions of England, as well as all nations of the UK. Dark blue nodes are observed.

Now for running our model not all observations are available at all levels. Hence we take a three stage approach to estimate the parameters as robustly as possible.

In first stage, Figure 1,we fit a model to entire of England, where we have all observations available, i.e., cases, deaths, ONS infections, REACT attack rate (total cumulative population infected). This means while fitting all four observations are used for inferring parameters namely via the observation process defined in Eq (1), Eq (5), Eq (9), and Eq (12). This step is very crucial as it is the only place we have an observation for infections. Hence, we estimate $ifr(w)$ and $iar(w)$ only in this step, in all other steps we use provide tight priors around the estimates from this step.

In second stage, Figure 1, we fit individual models to all regions in England, as well as to all nations in the UK. The observations used in this stage are deaths and cases, hence the likelihood of model in this stage is calculated by adding Eq (1) and Eq (5). As stated earlier the $ifr(w)$ and $iar(w)$ used in Eq (1) and Eq (5) are provided strong priors centred around the values estimated in first stage.

Finally, in our last stage, Figure 2, we fit individual models to all local authorities (LTLAs in England, local authorities in all other nations of the UK). As in second stage the likelihood of model is only composed of Eq (1) and Eq (5). Additionally in this stage we use the R_t of the region (region for LTLAs in England and nations for local authorities in other nations) as a covariate for R_t of the local area in addition to the weekly random walk.

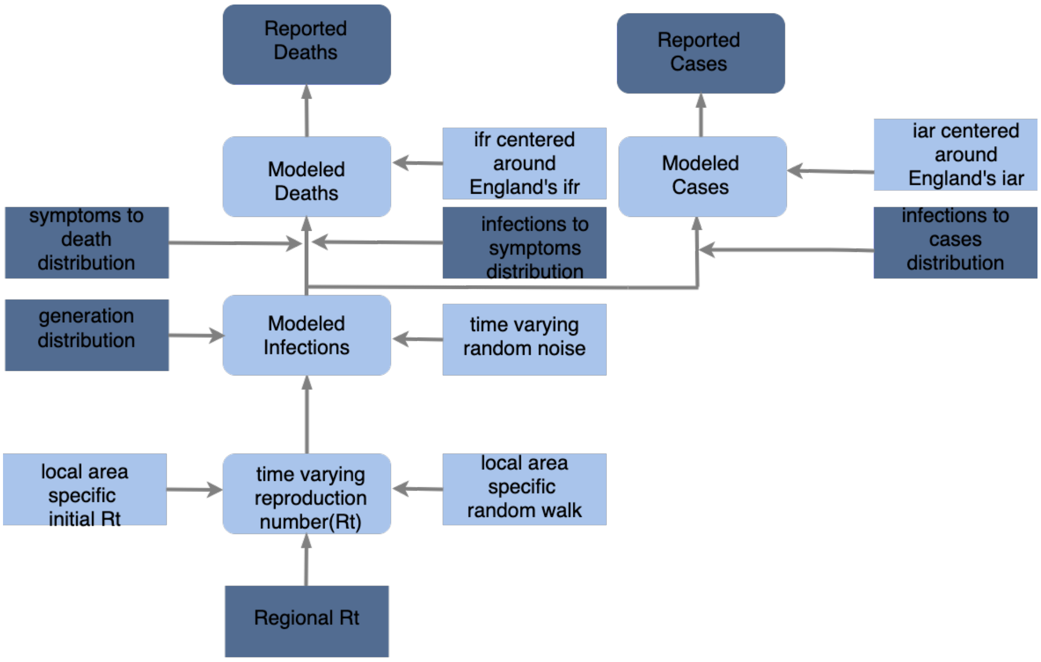

Fig. 2. Model diagram for local authorities epidemics modelling. Dark blue nodes are observed.

References

Bhatt, S., Ferguson, N., Flaxman, S., Gandy, A., Mishra, S. and Scott, J. A. (2020) Semi-Mechanistic Bayesian Modeling of COVID-19 with Renewal Processes. *arXiv.*

Carpenter, B., Gelman, A., Hoffman, M. D., Lee, D., Goodrich, B., Betancourt, M., Brubaker, M., Guo, J., Li, P. and Riddell, A. (2017) *Stan*: A Probabilistic Programming Language. *Journal of Statistical Software*, **76**, 1–32.

Flaxman, S., Mishra, S., Gandy, A., Unwin, H. J. T., Mellan, T. A., Coupland, H., Whittaker, C., Zhu, H., Berah, T., Eaton, J. W., Monod, M., Imperial College COVID-19 Response Team, Ghani, A. C., Donnelly, C. A., Riley, S. M., Vollmer, M. A. C., Ferguson, N. M., Okell, L. C. and Bhatt, S. (2020) Estimating the effects of non-pharmaceutical interventions on COVID-19 in Europe. *Nature.*

Liu, Y., Gayle, A., Wilder-Smith, A. and Rocklöv, J. (2020) The reproductive number of COVID-19 is higher compared to SARS coronavirus. *Journal of Travel Medicine.*

Office for National Statistics (2020) Coronavirus (COVID-19) Infection Survey - Office for National Statistics. URL: `https://www.ons.gov.uk/peoplepopulationandcommunity/healthandsocialcare/conditionsanddiseases/datasets/coronaviruscovid19infectionsurveydata`.

REACT Study (2020) Real-time Assessment of Community Transmission findings. URL: `https://www.imperial.ac.uk/medicine/research-and-impact/groups/react-study/real-time-assessment-of-community-transmission-findings`.

Riley, S., Ainslie, K. E. C., Eales, O., Jeffrey, B., Walters, C. E., Atchison, C. J., Diggle, P. J., Ashby, D., Donnelly, C. A., Cooke, G., Barclay, W., Ward, H., Taylor, G., Darzi, A. and Elliott, P. (2020) Community prevalence of SARS-CoV-2 virus in England during may 2020: REACT study. *medRxiv*.

Scott, J. A., Gandy, A., Mishra, S., Unwin, J., Flaxman, S. and Bhatt, S. (2021) epidemia: Modeling of epidemics using hierarchical Bayesian models. URL: https://imperialcollegelondon.github.io/epidemia/. R package version 1.0.0.

Verity, R., Okell, L. C., Dorigatti, I., Winskill, P., Whittaker, C., Imai, N., Cuomo-Dannenburg, G., Thompson, H., Walker, P. G. T., Fu, H., Dighe, A., Jamie, T., Gaythorpe, K., Green, W., Hamlet, A., Hinsley, W., Laydon, D. and Nedjati, G. (2020) Estimates of the severity of COVID-19 disease. *Lancet Infect Dis*.

Walker, P. G., Whittaker, C., Watson, O. J., Baguelin, M., Winskill, P., Hamlet, A., Djafaara, B. A., Cucunubá, Z., Mesa, D. O., Green, W. et al. (2020) The impact of COVID-19 and strategies for mitigation and suppression in low-and middle-income countries. *Science*, **369**, 413–422.
